# Leaf Volatile Compounds and Associated Gene Expression during Short-Term Nitrogen Deficient Treatments in *Cucumis* Seedlings

**DOI:** 10.3390/ijms17111713

**Published:** 2016-11-02

**Authors:** Jie Deng, Hong-Jun Yu, Yun-Yun Li, Xiao-Meng Zhang, Peng Liu, Qiang Li, Wei-Jie Jiang

**Affiliations:** 1 Key Laboratory of Horticultural Crops Genetic Improvement (Ministry of Agriculture), Institute of Vegetables and Flowers, Chinese Academy of Agricultural Sciences, Beijing 100081, China; 115425193@163.com (J.D.); yuhongjun@caas.cn (H.-J.Y.); yijinlidy@163.com (Y.-Y.L.); 13716247546@163.com (X.-M.Z.); pengliucaas@foxmail.com (P.L.); 2 College of Forestry and Horticulture; Xinjiang Agricultural University, Urumqi 830052, China

**Keywords:** nitrogen deficiency, cucumber, leaf volatile, aldehyde, LOX-HPL pathways

## Abstract

Nitrogen (N) is an important macronutrient for plant growth and development, but the regulatory mechanism of volatile compounds in response to N deficiency is not well understood, especially in cucumber, which consumes excessive N during growth. In this study, the major volatile compounds from cucumber leaves subjected to N deficiency were analyzed by GC-MS. A total of 24 volatile components were identified including 15 aldehydes, two ketones, two alkenes, and five other volatile compounds in 9930 leaves. Principal component analysis using volatile compounds from cucumber leaves provided good separation between N-sufficient and N-deficient treatments. The main volatiles in cucumber leaves were found to be C6 and C9 aldehydes, especially (*E*)-2-hexanal and (*E*,*Z*)-2,6-nonadienal. (*E*)-2-hexanal belonged to the C6 aldehyde and was the most abundant compound, whereas (*E*,*Z*)-2,6-nonadienal was the chief component of C9 aldehydes. During N-deficient treatment, short-chain volatile content was significantly improved at 5 day, other volatiles displayed significant reduction or no significantly changes in all sampling points. Improvement of short-chain volatiles was confirmed in the six other inbred lines at 5 day after N-deficient treatments. The expression analysis of 12 cucumber *LOX* genes and two *HPL* genes revealed that *CsLOX19*, *CsLOX20*, and *CsLOX22* had common up-regulated expression patterns in response to N-deficient stress in most inbred lines; meanwhile, most sample points of *CsHPL1* also had significant up-regulated expression patterns. This research focused on the relationship between volatiles in cucumber and different nitrogen environments to provide valuable insight into the effect of cultivation and management of the quality of cucumber and contributes to further research on volatile metabolism in cucumber.

## 1. Introduction

Cucumber (*Cucumis sativus* Linn.) is a widely grown vegetable with a fresh and distinct flavor [1]. During cucumber cultivation, excessive nitrogen (N) fertilizer application is used to achieve a high yield, but it also leads to substantial environmental degradation [2]. N fertilizer has been the major cost in cucumber production and reduces the income of farmers [3]. Furthermore, excessive application of N can deteriorate vegetable flavor [4] and decrease plant resistance to pathogens [5,6]. During N-deficient treatments, the antioxidant enzyme system (Superoxide dismutase, peroxidase, and catalase) of cucumber seedlings was activated markedly and exhibited higher levels of antioxidants [3,7]. Thus far, researchers have identified 78 volatile compounds from cucumber, including aldehydes, alcohols, esters, alkanes, furfurans, and others [8]. Volatile C6 and C9 aldehydes are considered important factors to the characteristic flavors of green leaves, fruits and vegetables. Plants produce volatile compounds, such as C6 aldehydes, C9 aldehydes, and their acetates, through the lipoxygenase (LOX) and hydroperoxidelyase (HPL) pathways [9]. Linoleic and α-linolenic acid are the substrates for dioxgenation and subsequent cleavage to obtain C6 and C9 volatile aldehydes, which can be further modified by alcohol dehydrogenases (ADH) [10,11]. (*E*,*Z*)-2,6-nonadienal (which has a cucumber-like flavor) and (*E*)-2-hexenal (which has an apple-like flavor) are the main volatile compounds [8,12]. Green leaf volatiles (GLVs) have also been reported to play important roles in different biological processes [13,14,15]. Contents of GLVs display improvement after infection with bacteria and pathogenic fungi [16,17,18], which indicates that a possible physiological function of these short-chain volatiles is to limit pathogen growth. Moreover, pretreatment with exogenous (*E*)-2-hexenal resulted in increased resistance against pathogens, most likely as a result of both activation of defense responses and direct inhibition of fungal growth [17,19]. In higher plants, short-chain aldehydes are also produced in response to wounding, and they also have important functions during wound healing and pest resistance [20]. Some GLVs were also demonstrated to be signal chemicals, which are produced by environmental stress to induce abiotic-related gene expression as oxidative stress signals [21].

Most prior research has focused on the types of volatile compounds present in cucumber fruit and their potential role in fruit development [8,22]. Little information is available about the mechanisms of volatile biosynthesis in cucumber seedlings. In the present study, the effect of nitrogen deficiency on volatile compounds in cucumber leaves was investigated on transcriptional and metabolic level, respectively. These results provide a basis for further studies on the regulation of C6 and C9 volatiles in cucumber leaves.

## 2. Results and Discussion

### 2.1. Analysis of Cucumber Leaf Volatiles by GC-MS and Variation in Total Volatile Content

**Figure 1:**
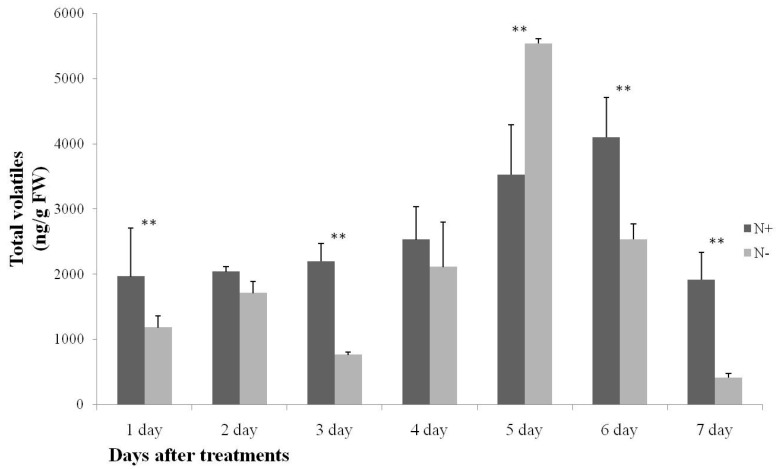
Changes of total volatile content in 9930 leaves during N-sufficient and N-deficient treatments. N+ and N− mean nitrogen sufficient and nitrogen deficient treatments, respectively. Bars present means ± standard deviations, data were analyzed by one-way ANOVA followed by the Duncan’s multiple range tests to make comparisons within both treatments in same day. Asterisks indicate significant difference, ** *p* < 0.01. Values are means of three replicates.

In the present study, we conducted a detailed volatile investigation of the metabolic response of cucumber leaves to N-sufficient and N-deficient treatments. The qualitative compounds were quantified and are listed in Table S1. A total of 24 volatile compounds were identified and quantified including 15 aldehydes, two ketones, two alkenes and five other volatile compounds. The amount of total volatiles accounted for 1914.71 to 4097.54 ng/g FW (fresh weight) in N-sufficient treatments and 411.73 to 5523.84 ng/g FW in N-deficient treatments. The lowest amount was detected at Day 7 during both treatments, and the highest content was detected at Days 6 and 5. At Days 1, 3, 6, and 7, the total amount of volatiles in N-deficient nutrient solution was significantly lower than in plants which were cultivated in N-sufficient nutrient solution. The total volatile content in the N-deficient treatment clearly increased at Day 5 (Figure 1).

A wide variation in leaf volatile compounds was observed among the different nitrogen treatments (Table S1). During N-deficient treatments, all the volatile compounds displayed a significant difference compared to the N-sufficient treatments from Days 1 to 7. Among the 24 volatile compounds, most showed lower level of volatile compounds in N-deficient treatments from Day 1 to Day 7, with the exception of hexanal, (*E*)-2-hexenal, and (*E*,*E*)-2,4-hexadienal which belong to the C6 aldehydes and displayed higher levels at Days 5, 2 and 3, respectively (Figure S1). Interestingly, β-caryophyllene and α-curcumene, which were detected in all N-sufficient leaves, were not found in any of the leaves from the N-deficient treatments.

### 2.2. Principal Component Analysis of Volatiles during N-Sufficient and N-Deficient Treatments

**Figure 2:**
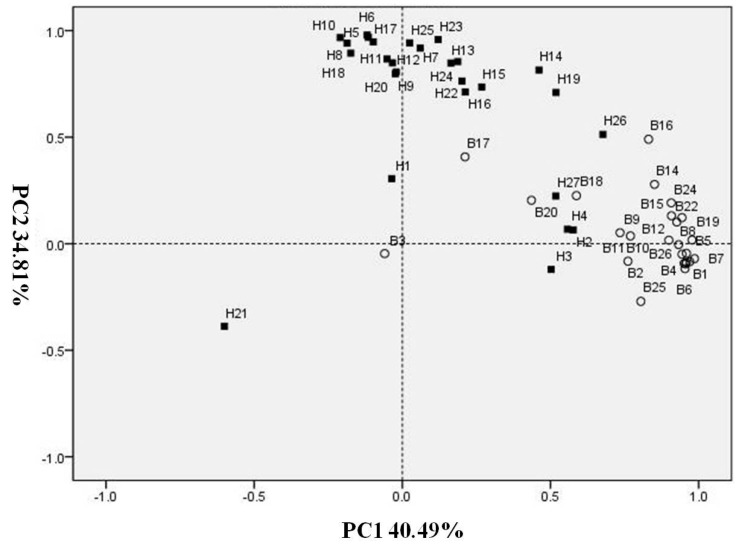
PCA of N-sufficient and deficient treatments based on the first 2 PCA results from volatile compounds. (**H**) Volatiles from N-sufficient treatments; (**B**) Volatiles from N-deficient treatments: **1**, Hexanal; **2**, (*E*)-2-Hexenal; **3**, (*E*,*E*)-2,4-Hexadienal; **4**, total C6 aldehydes; **5**, (*Z*,*Z*)-3,6-nonadienal; **6**, (*Z*)-6-Nonenal; **7**, Nonanal; **8**, (*E*,*E*)-2,6-Nonadienal; **9**, (*E*,*Z*)-2,6-Nonadienal; **10**, (*E*)-2-Nonenal; **11**, Total C9 aldehydes; **12**, Benzaldehyde; **13**, (*E*,*E*)-2,4-Heptadienal; **14**, Acetophenone; **15**, (*E*,*E*)-3,5-Octadien-2-one; **16**, 4-Heptenal; **17**, Linalool; **18**, Methyl salicylate; **19**, β-Cyclocitral; **20**, Undecanal; **21**, β-Caryophyllene; **22**, β-Ionone; **23**, α-Curcumene; **24**, β-Ionone epoxide; **25**, Tetradecanal; **26**, (9*E*,12*E*,15*E*)-9,12,15-Octadecatrienal; **27**, Total volatiles.

Using Principal Component Analysis (PCA) to examine the data, PCA scores plots allow visual representation of any separation of cucumber leaf volatiles when comparing the N-sufficient and N-deficient treatments. In the present study, the complete data set was selected for PCA and was performed on an average content of all compounds from N-sufficient and N-deficient treatments. Principal Component 1 (PC1) and PC2 accounted for approximately 75.30% of the total variability. More specifically, PC1 accounted for 40.49%, and PC2 was responsible for 34.81% (Figure 2).

Based on the loadings for this PCA, most volatiles of cucumber leaves grown in N-sufficient nutrition had higher concentration. When all volatiles of both N-sufficient and N-deficient treatments were combined, PCA using cucumber leaf composition parameters provided good separation (Figure 2). PCA on the profiles of volatile compounds resulted in a clustered pattern in which volatiles of the N-deficient treatment were mainly clustered in the positive axis of PC1 and volatiles of N-sufficient treatment were mainly clustered in the positive axis of PC2, with the exception of (*E*)-2-hexenal, (*E*,*E*)-2,4-hexadienal, total C6 aldehydes, and total volatiles, which displayed significant improvement in some points during N-deficient treatment. These results indicated that nitrogen supply affected the production of most cucumber leaf volatiles.

### 2.3. Changes of C6 and C9 Aldehyde Content during N-Deficient Treatment

**Figure 3:**
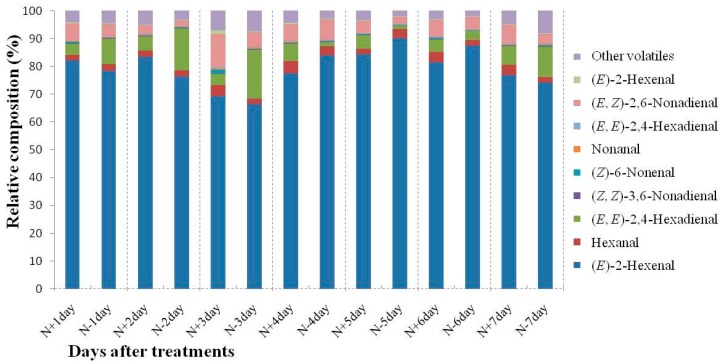
Relative composition (%) of volatiles from 9930 leaves of N-sufficient and deficient treatments. N+ and N− mean nitrogen sufficient and nitrogen deficient treatments, respectively. Values are means of three replicates.

C6 and C9 aldehydes are known to contribute to the flavor and aroma of plants and resistance to pathogens and are powerful inducers of abiotic stress-related genes [8,10,23]. C6 aldehydes provide a more “green grass” flavor, while C9 aldehydes enhance the unique “cucumber-like” flavor [22]. In this study, C6 and C9 aldehydes constitute the majority of the cucumber leaf volatiles (Figure 3.); three C6 aldehydes were detected in cucumber leaves including hexanal, (*E*)-2-hexenal, and (*E*,*E*)-2,4-hexadienal. The proportion of these C6 compounds to total volatiles accounted for 77.96% to 91.11% in N-sufficient treatments and 86.05% to 94.94% in N-deficient treatments. Most of the relative composition of C6 aldehydes in N-deficient treatments is higher than those that were N-deficient, except on 7 day. The same trends of the C6 aldehyde ratio “low-high-low-high-low” pattern were observed during N-sufficient and N-deficient treatments; this might be caused by difference of environment. Of the three C6 aldehydes, (*E*)-2-hexanal had the highest relative proportion, which accounted for 69.05% to 84.05%.

Six C9 aldehydes were detected in cucumber leaves, including (*E*,*Z*)-2,6-nonadienal, (*E*,*E*)-2,6-nonadienal, (*Z*,*Z*)-3,6-nonadienal, (*Z*)-6-nonenal, nonanal, and (*E*)-2-nonenal. The relative proportion of C9 aldehydes accounted for 4.04% to 15.87% during N-sufficient treatments and for 2.84% to 8.42% during N-deficient treatments. Among the six C9 aldehydes, (*E*,*Z*)-2,6-nonadienal had the highest proportion and accounted for 2.06% to 12.06% in all sample points. At four days after N-deficient treatments, the C9 relative composition was higher than cultivated in N-sufficient treatments, while the other six sampling points displayed lower values than controls. During N-deficient treatments, the relative composition of C6 and C9 aldehydes were varied, the relative composition of C6 aldehydes was improved, and the amount of C9 aldehydes was reduced for most sample points.

**Figure 4:**
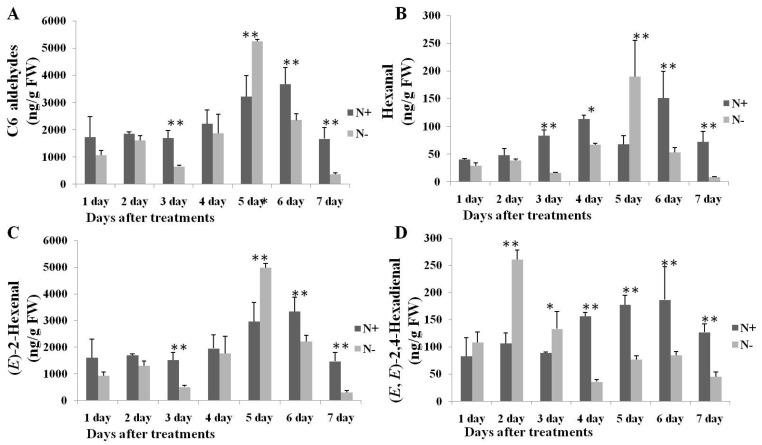
Changes in content of: C6 aldehydes (**A**); hexanal (**B**); (*E*)-2-hexenal (**C**); and (*E*,*E*)-2,4-Hexadienal (**D**) in 9930 leaves during N-deficient treatments. Bars present means ± standard deviations. * and ** mean significant difference from control at *p* < 0.05 and *p* < 0.01, respectively. N+ and N− mean nitrogen sufficient and nitrogen deficient treatments, respectively. Values are means of three replicates.

Compared to N-sufficient treatments, content of (*E*,*E*)-2,4-hexadienal displayed significant improvement at 2 and 3 day, hexanal and (*E*)-2-hexenal were also significantly improved at 5 day. (Figure 4). The amount of total C6 aldehydes ranged from 1696.69 to 3679.16 ng/g FW during the N-sufficient treatments and from 358.87 to 5255.86 ng/g FW during N-deficient treatments. The amount of (*E*)-2-hexenal reached a maximum value of 4989.15 ng/g FW on Day 5. During N-deficient treatments, the amount of (*E*)-2-hexenal and hexanal displayed a decrease compared to N-sufficient treatments, with the exception of Day 5, on which a significant increase was displayed. The content of (*E*,*E*)-2,4-hexadienal displayed a different pattern during N-deficient treatment, increasing from Day 1 to Day 3 and decreasing from Day 4 to Day 7.

**Figure 5:**
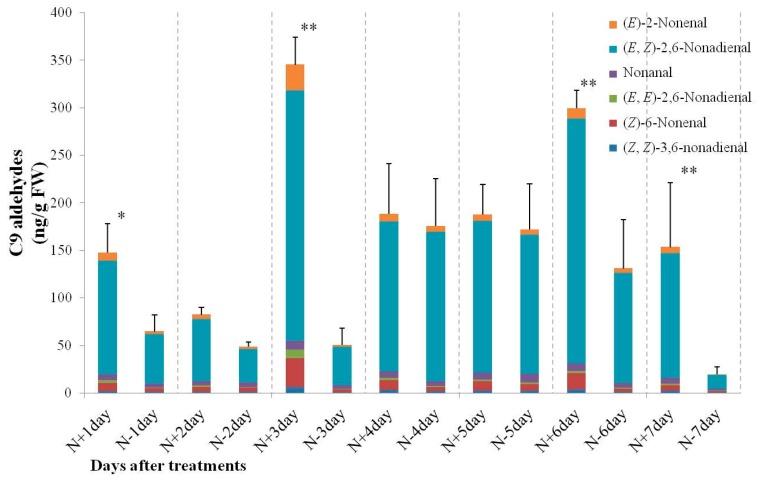
Changes in content of C9 aldehydes in 9930 leaves during N-sufficient and deficient treatments. Bars present means ± standard deviations. * and ** mean significant difference from control at *p* < 0.05 and *p* < 0.01, respectively. N+ and N− mean nitrogen sufficient and nitrogen deficient treatments, respectively. Values are means of three replicates.

During N-deficient treatments, the amount of C9 aldehydes also displayed significant changes compared to the control. As shown in Figure 5, the total amount of C9 aldehydes in N-deficient treatments was significantly reduced at Days 1, 3, 6, and 7. During the N-sufficient and N-deficient treatments, the highest amounts of C9 aldehydes were exhibited at Days 3 and 4 with 345.11 and 175.68 ng/g FW, respectively. (*E*,*Z*)-2,6-nonadienal was the main component of C9 aldehydes, accounting for 70.13% to 88.74% of the total C9 aldehydes. A higher ratio of (*E*,*Z*)-2,6-nonadienal between N-sufficient and N-deficient treatments was observed on Days 4 and 5, but no significant difference was observed. According to these results, we inferred that nitrogen acts as the key signal involved in the regulation of the short-chain volatile biosynthesis, not only for the amounts but also for the relative composition, and the amount of C6 aldehydes could be increased in response to N-deficiency.

### 2.4. Relationship between C6 and C9 Aldehydes and Gene Expressions in the 9930 Line

**Figure 6:**
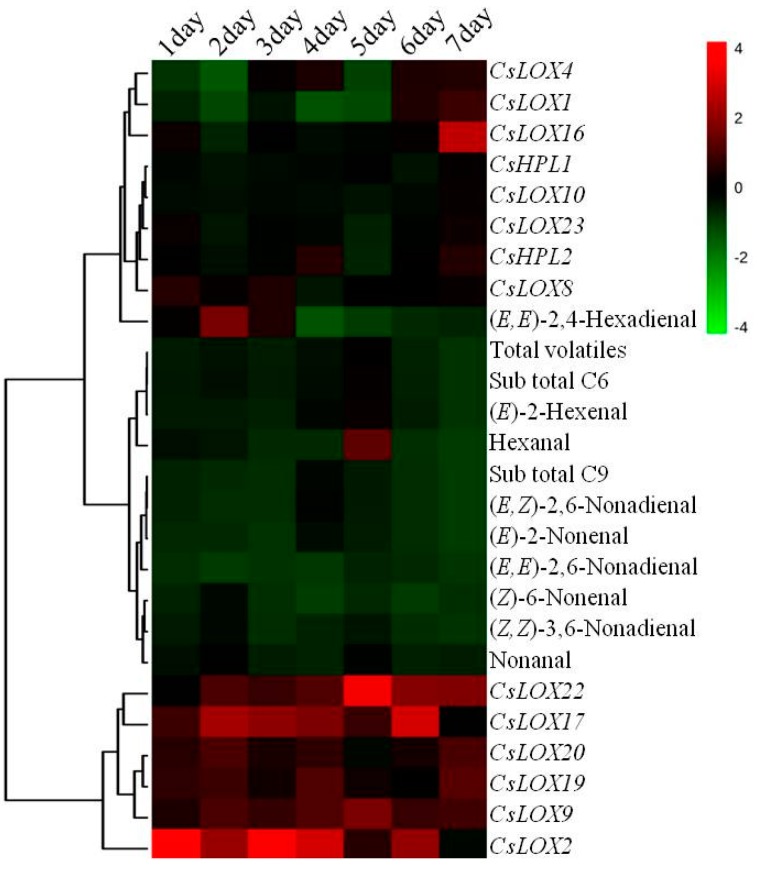
Hierarchical cluster dendrogram and heat map of volatiles content and gene expression folds during N-sufficient and deficient treatments in 9930 leaves. Leaves were sampled at 1, 2, 3, 4, 5, 6 and 7 day after N-deficient treatments. The heat map has been generated based on the fold-change values of volatile contents, *CsLOXs* and *CsHPLs* expression level in the treated samples when compared with its unstressed control samples by MetaboAnalyst 3.0 software. The color scale for fold-change values is shown on the right. Values of gene expression folds are means of three biological replicates with three technical replicates. Values of short-chain volatiles are means of three biological replicates.

In order to explore potential correlations between aldehydes, the folds of *CsLOXs* and *CsHPLs* gene expression patterns were used for HCA analysis, and N-deficient treatments were compared to N-sufficient treatments. HCA data resulted in two distinct sample clusters (Figure 6). Cluster I included six *CsLOXs* (*CsLOX2*, *9*, *17*, *19*, *20*, and *22*) that displayed higher up-regulated expression patterns during N-deficient treatments. Cluster II included all nine aldehydes, six *CsLOXs*, and two *CsHPLs*. The expression of *CsHPL1* was significantly increased at 5 d, meanwhile the amount of (*E*)-2-hexenal and hexanal were also significantly up-regulated. This result indicated that *CsLOX2*, *9*, *17*, *19*, *20*, and *22* were responsive to N-deficiency and had higher up-regulated expression patterns during short-term N-deficient treatments, thus the increase in C6 aldehydes might be induced by the up-regulation of *CsHPL1*.

### 2.5. Changes of C6 and C9 Volatile Content during N-Deficient Treatment in Six Other Inbred Cucumber Lines

**Figure 7:**
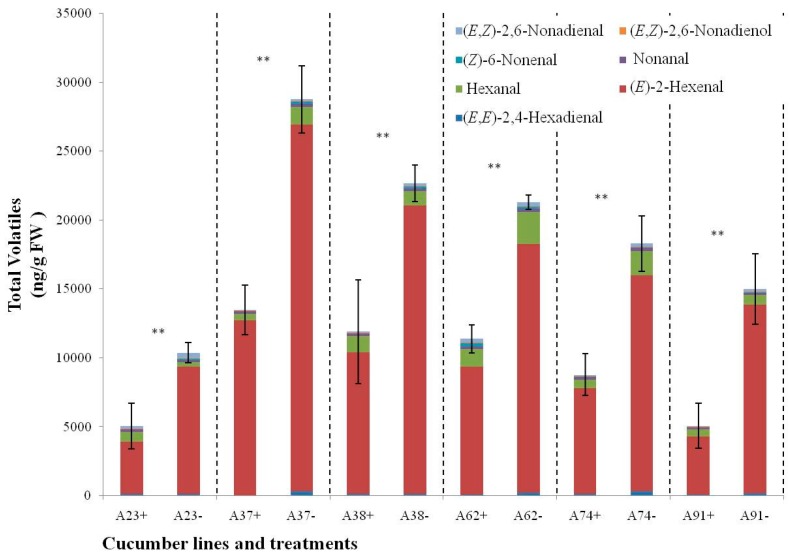
Changes in content of short-chain volatiles in six inbred cucumber leaves at 5 day after N-deficient treatments. Bars present means ± standard deviations. + and − mean N-sufficient and deficient treatments, respectively. ** mean significant difference from control at *p* < 0.01. Values are means of three biological replicates.

In order to further study the response of the C6 and C9 volatile metabolism to N-deficient treatments, six other inbred cucumber lines were cultivated in N-deficient nutrition solution and sampled at Day 5 for detecting leaf volatiles. Seven short-chain leaf volatiles (hexanal, (*E*,*E*)-2,4-hexadienal, (*E*)-2-hexenal, nonanal, (*Z*)-6-nonenal, (*E*,*Z*)-2,6-nonadienal, and (*E*,*Z*)-2,6-nonadienol) were detected in cucumber leaves, as shown in Figure 7 and Table S3, compared to N-sufficient treatments, the total amount of short-chain volatiles of all six lines was significantly improved in the N-deficient treatments, up-regulation of 0.87- to 1.96-fold was displayed, and the amount of C6 volatiles of all the six lines and C9 volatiles of five lines increased. Of the six inbred lines, (*E*)-2-hexenal was still the main component, displaying as much as 75.38% in all treatments. (*E*,*Z*)-2,6-nonadienol was the smallest component, displaying between only 0.02% and 0.73% in all treatments. During N-deficient treatments, changes in volatile components were observed among six inbred lines: 32 volatiles were up-regulated and 10 volatiles were down-regulated at 5 day. All of the (*E*)-2-hexenal amounts, which had green-like flavor, as well as (*E*,*Z*)-2,6-nonadienal, which had cucumber-like flavor, were improved compared to N-sufficient treatments. The other five volatiles displayed down-regulation in some lines. Among the six inbred lines, different responses to N-deficiency were also observed. For A37 and A91, the amount of all seven volatiles increased. For A74, only one volatile decreased, but for A23, the content of (*E*)-2-hexenal, (*E*,*Z*)-2,6-nonadienol, and (*E*,*Z*)-2,6-nonadienal displayed up-regulation, whereas the amount of the other four volatiles decreased.

Combined with the changes of 9930 and the volatile content of six other inbred lines, we suggest that the amount of short-chain leaf volatiles in cucumber seedlings could be significantly improved in response to nitrogen deficiency. A previous study showed that reactive short-chain leaf volatiles (RSLVs) such as (*E*)-2-hexenal can act as signal chemicals that strongly induce the gene expression of abiotic-related transcription factors. (*E*)-2-hexenal could up-regulate genes that respond to salt, oxidation, osmotic drought, cold, and wounding [21]. In the present study, RSLVs might involve in the induction of the expression of down-stream transcription factors during nitrogen starvation.

### 2.6. The Expression Patterns of Cucumber LOX Genes in Response to N-Deficient Treatments in Six Inbred Cucumber Lines

LOX proteins and their metabolites have been demonstrated to involve in production of short-chain volatile compounds [11] and plant defense responses during various stresses [24,25]. To study the effect of nitrogen deficiency on the biosynthesis of volatiles in cucumber seedlings, *LOX* gene expression profiles in cucumber leaves were analyzed by qPCR.

Of the 12 *LOX* genes, six *CsLOXs* (*CsLOX1*, *2*, *4*, *8*, *9*, and *10*) were predicted to be type-1 *LOXs*, which are involved in the biosynthesis of C9 aldehydes. The other six *LOXs* (*CsLOX16*, *17*, *19*, *20*, *22*, and *23*) were predicted to be type-2, which are involved in the biosynthesis of C6 aldehydes [23]. In our study, compared to N-sufficient treatments in six inbred cucumber lines, 157 of 276 sample points of *CsLOXs* expressed higher levels during N-deficient treatments (Table S2), including 68 type-1 *CsLOXs* and 89 type-2 *CsLOXs* sample points. Two types of *CsLOXs* displayed a clear difference in response to N-deficiency treatments, and type-2 *CsLOXs* had more up-regulated expression patterns. Of the six inbred lines, different expression patterns of *CsLOXs* were observed during N-deficient treatments. For A23, *CsLOX9* had higher up-regulated patterns; for A74 and A91, *CsLOX19, 20*, and *22* displayed higher up-regulation. For A38, *CsLOX10* displayed up-regulation (Table S2).

**Figure 8:**
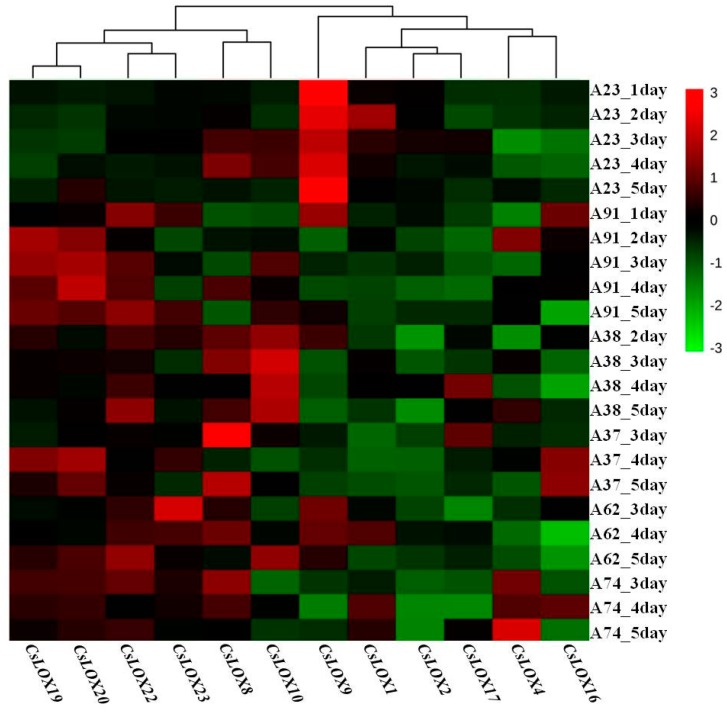
Hierarchical cluster dendrogram and heatmap of *CsLOXs* expression folds in six inbred cucumber leaves during N-deficient treatments. The heat map has been generated based on the fold-change values of *CsLOXs* expression level in the treated samples when compared with its unstressed control samples. The color scale for fold-change values is shown at the right. Values are means of three biological replicates with three technical replicates.

HCA was employed to explore potential correlations among 12 *CsLOXs*, and, to stratify samples based on trait, Ward’s linkages in Euclidian distance were used. HCA data resulted in two distinct sample clusters (Figure 8). Cluster I included 6 *CsLOXs* (*CsLOX8*, *10*, *19*, *20*, *22*, and *23*) which displayed more up-regulated expression patterns during N-deficient treatments. The six other *CsLOXs* belonged to Cluster II and displayed more down-regulated patterns.

A previous study showed that *LOXs* were involved in the response to abiotic stress and plant growth regulators [11]. Our results from the expression of *CsLOXs* demonstrated that type-1 and type-2 *CsLOXs* had different response patterns to short term N-deficiency. In most sample points during N-deficient treatments, type-1 *CsLOXs* were down-regulated and type-2 *CsLOXs* were up-regulated. Combined with the expression patterns of the 9930 line, we suggest that *CsLOX19*, *CsLOX20*, and *CsLOX22* which belong to type-2 *CsLOXs* might be involved in the response to N-deficiency and the improvement of C6 aldehydes content might be partly caused by the up-regulation of type-2 *CsLOXs* in cucumber leaves.

### 2.7. The Expression Patterns of CsHPLs during N-Deficient Treatments in Six Inbred Cucumber Lines

In the LOX-HPL pathway, HPL is one of the key enzymes that catalyzes 13-HPOD/HPOT or 9-HPOD/HPOT to produce short-chain volatiles. In cucumber, three *HPL* genes were divided into three types by their specificities of substrate: 13-HPL which specifically catalyzes 13-HPOD/HPOT to produce C6 and C12 volatiles, 9-HPL which catalyzes 9-hydroperoxides to form C9 aldehydes, and 9/13-HPL, which can use both 9- and 13-hydroperoxides as substrates to produce C6 and C9 aldehydes [22,25]. To study the changes in the expression patterns of *CsHPLs* during N-deficient treatments, *HPLs* gene expression profiles in cucumber leaves were analyzed by qPCR.

**Figure 9:**
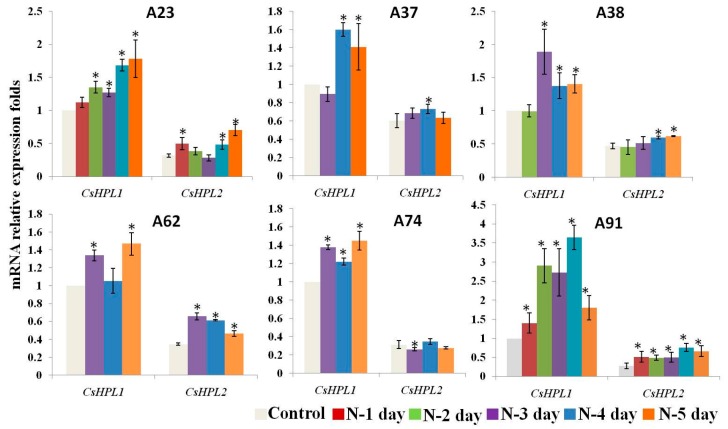
Expression profiles of *HPL* genes in six inbred cucumber leaves during N-deficient treatments. Leaves were sampled at 1, 2, 3, 4 and 5 d after N-deficient treatments. The map has been generated based on the fold-change values of *CsHPLs* in the treated samples when compared with its unstressed control samples. The *CsHPL1* expression level of control was considered as one-fold. Bars present means ± standard deviations. * means significant difference from control at *p* < 0.05. Values are means of three biological replicates with three technical replicates.

In our study, only *CsHPL1* (13-HPL) and *CsHPL2* (9/13-HPL) were expressed in cucumber leaves. For the six inbred lines, as shown in Figure 9, *CsHPL1* had a higher expression level than *CsHPL2* and most sample points of *CsHPL1* were significantly up-regulated during N-deficient treatments; no significantly reduced points were observed. For *CsHPL2*, 13 out of 23 sample points were significantly up-regulated during N-deficient treatments, and only one point was down-regulated significantly. For A91, all the sample points of *CsHPL1* and *CsHPL2* were significantly up-regulated compared to N-deficient treatments.

Our results show that content of short-chain volatiles was significantly improved at Day 5; meanwhile, the expression pattern of *CsHPL1* was also significantly improved in all seven inbred lines. A previous study showed that C9 aldehydes are the major volatiles in cucumber fruit [26]. The loss of 9-HPL enzyme activity and the fact that 9/13-HPL enzyme preferentially exhibits 13-HPL enzyme activity [22] in cucumber leaves might be the main reasons for the relative composition difference of C6 and C9 aldehydes between cucumber fruit and leaves. According to these results, we suggest that the significant improvement of short-chain volatiles in cucumber was partly caused by the up-regulated expression of *CsHPL1* during N-deficient treatments.

## 3. Material and Methods

### 3.1. Plant Materials and N-Deficient Treatments

Cucumber (*Cucumis sativus*) cv. Lines 9930 were grown on 15 March 2015 and harvested on 23 April. The other six inbred lines (A23, A37, A38, A62, A74 and A91, cultivars of northern China) were grown 25 October and harvested on 10 December. Seeds were surface sterilized with 5% (*w*/*v*) NaClO for 15 min and germinated on wet filter paper in the dark. Cucumber plants were then moved into a sponge floating and cultivated in a hydroponic system containing modified Hoagland solution (11 mM NO_3_^−^, 1 mM NH_4_^+^, pH 6.0) in a green house. The solutions were renewed every three or four days. After three true leaves reached, cucumber seedlings were moved into an N-free nutrient solution as N-deficiency treatment (0 mM NO_3_^−^, 0 mM NH_4_^+^; Ca(NO_3_)_2_ was replaced with CaCl_2_; other ions were same as N-sufficient conditions except for chlorid ion (Table S4)), and plants cultivated in N-sufficient conditions as controls. The second fully expanded apical leaves were harvested and immediately frozen in liquid nitrogen and stored at −80 °C until analysis. Plant samples were taken with three biological replicates.

### 3.2. HS-SPME Extraction

Cucumber leaves were fully grinded in liquid nitrogen and then two hundred microgram sample was put into a glass vial, 0.4 mL of saturated sodium chloride solution which contained 50 ng of 2-Heptanonel as an internal standard were added to terminate enzymatic reaction and improve the volatiles into the headspace. After micro-extraction, volatiles were transferred into a head vial with crimp cap, and then fifty/thirty micromoles of CAR/DVB/PMDS fiber (Supleco, Bellefonte, PA, USA) were used for the next analysis. Samples were continuous agitated at 40 °C for 30 min (600 rpm), volatile compounds were also extracted under the same conditions.

### 3.3. GC-MS Analysis

Samples were analyzed by 7890A GC gas chromatograph combined with a 5975C mass spectrometer (Agilent Technologies, Santa Clara, CA, USA) on a DB5-MS capillary column (30 m × 0.25 mm × 0.25 µm with 5% Phenyl methyl siloxane, J & W Scientific, Folsom, CA, USA). GC condition was: helium was the carrier gas and flow rate through the column was 1.5 mL per min. The oven temperature programmed at 40 °C for 1.5 min, then ramped up to 150 °C at 5 °C per minute, after holding there for 13 min, ramped up to 230 °C at 15 °C per minute, and held for 15 min. Mass spectra were obtained at 70 eV.

### 3.4. RNA Isolation and qPCR

Total RNA was isolated from cucumber leaves of seedlings using the Trizol reagent (Invitrogen, Carlsbad, CA, USA) according to the manufacturer’s instructions. Concentration and purity of RNA were determined with a NanoDrop ND-1000 photospectrometer. cDNA was synthesized with 5 μg of total RNA using the M-MLV reverse transcriptase (Promega, Madison, WI, USA). The specific primers of 23 *CsLOX* genes were synthesized by previous study [11]. Three cucumber *HPL* gene sequences were downloaded from Cucumber Genome DataBase [27], the specific primers were designed using DNAMAN 6.0 (Lynnon Biosoft, San Ramon, CA, USA) for RT-PCR and qPCR (*CsHPL1*-F: TACCTTCATCTATTTCCCCC, *CsHPL1*-R: GAAGAACTTATCGGGTCCTT; *CsHPL2*-F: CCCGAATTACCAAATACAAC, *CsHPL2*-R: TAAGTTCCGTCGAGAATGTT; *CsHPL3*-F: TCATCTTCTTCAGAACACCC, *CsHPL3*-R: GATCGGAAGAAGGTTTCTCT). The amplified PCR products were quantified by an Applied BioSystems 7500 Real Time PCR System (Bio-Rad Laboratories, Hercules, CA, USA), with a SYBR Premix Ex Taq kit (Takara, Tianjin, China). All qPCR analyses were performed in three biological replicates with three technical replicates and the results were generated using 7500 software (Bio-Rad Laboratories, Hercules, CA, USA). For relative quantification, *CsACT* (Csa2M301530) (for the quantitation of gene expression during N-deficient treatments) gene was detected as an internal reference, and the 2^−ΔΔ*C*t^ method was used. PCR conditions were as follows: 5 min initial denaturation at 95 °C followed by 30 cycles of denaturation at 94 °C for 30 s, annealing at 50 °C for 30 s and an extension at 72 °C for 2 min. qPCR analysis was performed according to Zhao et al. [3].

### 3.5. Statistical Analysis

GC-MS analysis was performed according to Azam et al. [28], and the 2-Heptanone was used as internal standard for calculating content of volatiles. All data were expressed as the means ± SD of three replicates and used for multivariate analysis. Statistical analysis was performed using ANOVA within SPSS 22.0 (IBM, Armonk, NY, USA). Significant differences between leaves at different nitrogen treatments were confirmed using the Duncan's multiple range test. Principal component analysis (PCA) was used to detect clustering and to investigate possible relationships between different nitrogen treatments and volatile compounds. Heat map generation and hierarchical cluster analysis (HCA) were carried out using MetaboAnalyst 3.0 software [29].

## 4. Conclusions

Volatiles of cucumber leaves in 9930 and six other inbred lines were identified, and the expression patterns of 12 *CsLOXs* and two *CsHPLs* during nitrogen-deficient treatments were also detected by qPCR. The results indicate that the amount of short-chain volatiles was significantly improved during N-deficient treatments at 5 day, while other volatiles had no significant improvement at all sampling points. *CsLOXs* and *CsHPLs* displayed differential expression patterns in response to nitrogen deficiency. According to the results, we suggest that *CsLOX19*, *CsLOX20*, and *CsLOX22,* three type-2 LOXs predicted to be 13-LOX, and *CsHPL1* might be involved in response to N-deficiency and the increase of short-chain volatiles in cucumber leaves. The present work provides a valuable contribution toward the relationship between nitrogen and leaf volatiles and also enriches the databank of leaf volatiles. In addition, it can provide a more complete understanding of the LOX-HPL gene regulation patterns during nitrogen-deficient treatments.

## Supplementary Materials

Supplementary materials can be found at www.mdpi.com/1422-0067/17/11/1713/s1.
