# Supplementary material for: Leaf Volatile Compounds and Associated Gene Expression during Short-Term Nitrogen Deficient Treatments in Cucumis Seedlings
Source: Int J Mol Sci. 2016 Nov 2;17(11):1713. doi: 10.3390/ijms17111713 (PMC5133771; doi:10.3390/ijms17111713)
Supplement: Supplementary file 1 [file ijms-17-01713-s001.pdf]

Supplementary Materials: Leaf Volatile Compounds and Associated Gene Expression during Short-Term Nitrogen Deficient Treatments in *Cucumis* Seedlings

Jie Deng, Hong-Jun Yu, Yun-Yun Li, Xiao-Meng Zhang, Peng Liu, Qiang Li and Wei-Jie Jiang

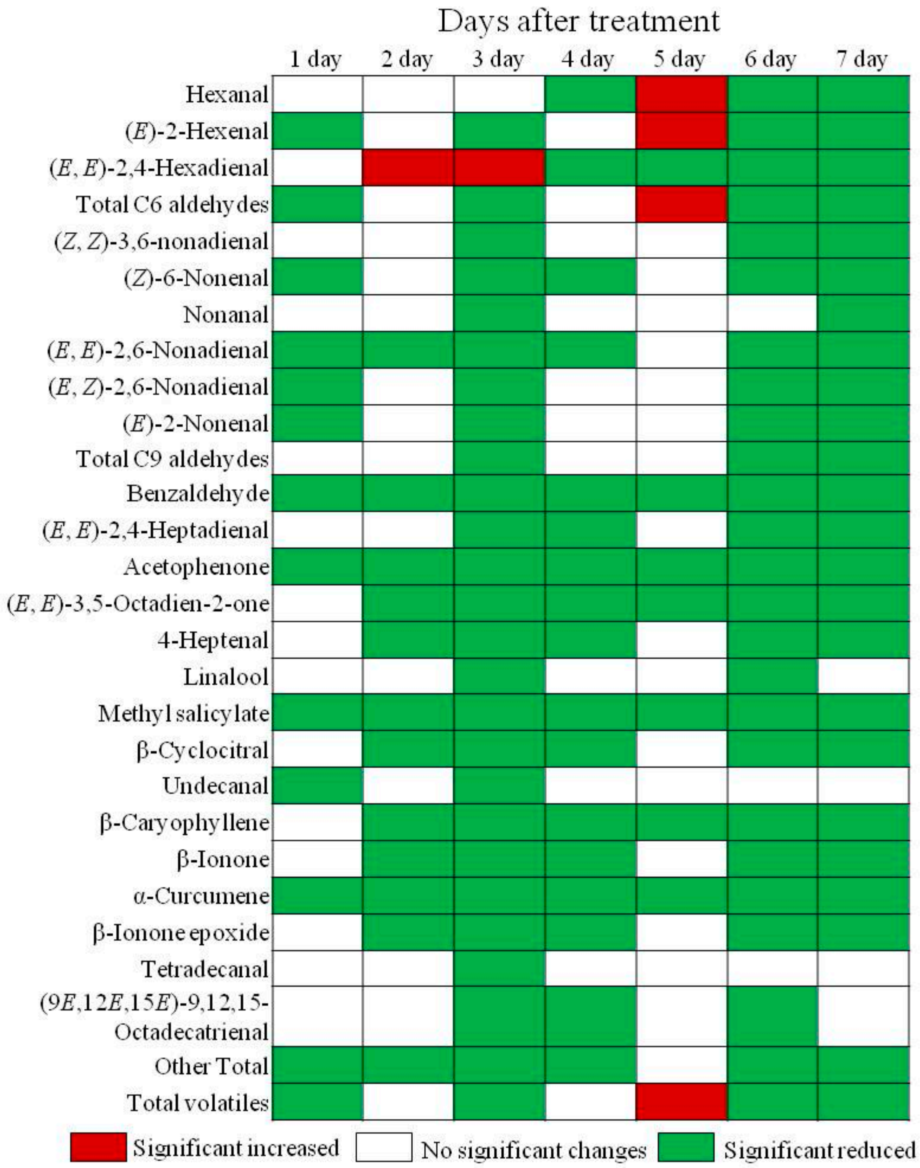

Figure S1. Changes of volatile compounds in 9930 leaves during N-sufficient/deficient treatments.

Table S1. Identification and quantification of volatiles during N-sufficient/deficient treatment in cucumber leaves.

Compounds Name	Nitrogen Sufficient Treatments						
	1 Day	2 Day	3 Day	4 Day	5 Day	6 Day	7 Day
Hexanal	40.11 ± 2.2	47.72 ± 12.2	83.5 ± 9.86	113.04 ± 7.57	67.73 ± 15.78	151.22 ± 48.58	71.79 ± 19.36
(*E*)-2-Hexenal	1610.17 ± 698.97	1698.55 ± 46.14	1523.9 ± 282.5	1958.34 ± 498.42	2972.1 ± 715.18	3341.22 ± 533.55	1471.49 ± 337.58
(*E*,*E*)-2,4-Hexadienal	82.92 ± 34.07	106.5 ± 19.52	89.28 ± 1.33	156.58 ± 7.24	177.4 ± 17.97	186.72 ± 60.53	127.25 ± 14.95
Sub total C6	1733.2 ± 734.97	1852.78 ± 62.35	1696.69 ± 293.39	2227.95 ± 506.85	3217.24 ± 747.79	3679.16 ± 601.31	1670.53 ± 367.18
(*Z*,*Z*)-3,6-nonadienal	1.88 ± 0.33	1.31 ± 0.12	6.01 ± 1.54	2.91 ± 0.83	2.7 ± 0.25	3.53 ± 1.67	2.46 ± 1.11
(*Z*)-6-Nonenal	8.34 ± 1.62	4.59 ± 1.01	30.4 ± 5.95	10.19 ± 3.63	9.18 ± 1.46	17.14 ± 4.65	5.57 ± 0.22
Nonanal	5.75 ± 1.23	3.98 ± 1.05	9.42 ± 1.62	6.81 ± 1.09	6.81 ± 2.16	7.45 ± 3.15	5.61 ± 1.77
(*E*,*E*)-2,6-Nonadienal	3.32 ± 0.16	2.02 ± 0.56	8.84 ± 2.23	2.66 ± 0.41	2.31 ± 0.25	2.63 ± 1.08	1.91 ± 0.57
(*E*,*Z*)-2,6-Nonadienal	119.95 ± 26.75	66.08 ± 5	263.58 ± 22.36	157.96 ± 46.63	159.86 ± 25.15	257.74 ± 12	131.3 ± 62.93
(*E*)-2-Nonenal	8.48 ± 0.54	4.39 ± 1.14	26.85 ± 9.22	7.71 ± 0.4	6.87 ± 3.34	10.98 ± 1.8	6.6 ± 2.01
Sub total C9	147.71 ± 30.48	82.36 ± 7.94	345.11 ± 29.19	188.23 ± 52.77	187.74 ± 31.64	299.47 ± 18.93	153.45 ± 68
Benzaldehyde	22.79 ± 0.65	18.39 ± 4.13	26.58 ± 2.35	19.71 ± 0.98	19.21 ± 2.29	18.5 ± 2.27	16.45 ± 1.06
(*E*,*E*)-2,4-Heptadienal	34.3 ± 6.13	50.03 ± 21.42	79.21 ± 16.25	55.73 ± 20.37	61.84 ± 9	62.89 ± 8.8	48.36 ± 11.5
Acetophenone	3.54 ± 0.23	3.08 ± 0.57	3.98 ± 0.32	3.65 ± 0.16	3.84 ± 0.35	3.48 ± 0.65	3.15 ± 0.19
(*E*,*E*)-3,5-Octadien-2-one	3.2 ± 0.23	4.71 ± 2.01	7.72 ± 0.12	5.67 ± 1.69	6.26 ± 1.51	7.84 ± 0.99	3.79 ± 0.98
4-Heptenal	10.05 ± 3.29	16.93 ± 3.36	19.63 ± 8.24	15.86 ± 7.2	16.05 ± 1.37	14.43 ± 3.25	11.69 ± 1.93
Linalool	2.84 ± 0.78	1.54 ± 0.24	4.57 ± 0.21	2.36 ± 0.45	2.44 ± 0.49	2.65 ± 1.1	2.03 ± 0.89
Methyl salicylate	1.23 ± 0.02	0.9 ± 0.22	2.68 ± 0.31	1.02 ± 0.15	0.78 ± 0.09	0.83 ± 0.15	0.6 ± 0.09
β-Cyclocitral	0.5 ± 0.11	0.77 ± 0.46	1.29 ± 0.08	0.96 ± 0.36	1.12 ± 0.5	0.86 ± 0.2	0.52 ± 0.22
Undecanal	0.39 ± 0.08	0.26 ± 0.08	0.5 ± 0.06	0.28 ± 0.05	0.33 ± 0.07	0.32 ± 0.11	0.27 ± 0.05
β-Caryophyllene	0.15 ± 0	0.3 ± 0.28	0.53 ± 0.23	0.31 ± 0.04	0.23 ± 0.01	0.31 ± 0.04	0.24 ± 0.12
β-Ionone	1.57 ± 0.33	2.64 ± 1.22	5.35 ± 1.28	4.34 ± 0.53	4.15 ± 0.7	5.16 ± 1.95	2.64 ± 0.63
α-Curcumene	0.16 ± 0.04	0.12 ± 0.04	0.35 ± 0.06	0.15 ± 0	0.18 ± 0.07	0.15 ± 0.03	0.08 ± 0.03
β-Ionone epoxide	0.33 ± 0.1	0.53 ± 0.33	1.04 ± 0.32	0.74 ± 0.11	0.8 ± 0.15	0.91 ± 0.23	0.42 ± 0.13
Tetradecanal	0.25 ± 0.04	0.23 ± 0.08	0.62 ± 0.23	0.29 ± 0.06	0.38 ± 0.09	0.34 ± 0.08	0.31 ± 0.09
(9*E*,12*E*,15*E*)-9,12,15-Octadecatrienal	0.15 ± 0.03	0.13 ± 0	0.28 ± 0.07	0.28 ± 0.06	0.36 ± 0.08	0.26 ± 0.03	0.18 ± 0.07
Other Total	81.46 ± 9.41	100.55 ± 26.98	154.34 ± 29.52	111.34 ± 31.86	117.98 ± 12.7	118.91 ± 18.71	90.72 ± 14.29
Total	1962.37 ± 749.22	2035.69 ± 78.85	2196.13 ± 276.02	2527.52 ± 507.39	3522.95 ± 771.63	4097.54 ± 614.2	1914.71 ± 416.37

Table S1. *Cont.*

Compounds Name	Nitrogen Deficient Treatments						
	**1 Day**	**2 Day**	**3 Day**	**4 Day**	**5 Day**	**6 Day**	**7 Day**
Hexanal	29.22 ± 4.85	38.82 ± 1.91	16.58 ± 0.85	67 ± 2.87	189.76 ± 66	52.91 ± 9.04	8.68 ± 0.6
(*E*)-2-Hexenal	925.13 ± 148.41	1304.61 ± 176.49	506 ± 58.2	1772.24 ± 639.72	4989.15 ± 155.12	2215.04 ± 222.91	305.02 ± 62.04
(*E*,*E*)-2,4-Hexadienal	108.2 ± 19.51	260.94 ± 17.36	133.36 ± 32.14	36.04 ± 3.45	76.95 ± 6.37	84.97 ± 6.28	45.16 ± 8.4
Sub total C6	1062.56 ± 168.85	1604.37 ± 168.59	655.94 ± 26.97	1875.28 ± 640.85	5255.86 ± 112.38	2352.93 ± 218.68	358.87 ± 66.39
(*Z*,*Z*)-3,6-nonadienal	1.08 ± 0.25	1.24 ± 0.35	0.89 ± 0.26	1.93 ± 0.92	2.71 ± 0.84	1.5 ± 0.33	0.5 ± 0.1
(Z)-6-Nonenal	3.62 ± 1.55	4.26 ± 0.93	2.67 ± 0.72	4.16 ± 2.06	6.43 ± 2.22	2.81 ± 0.11	1.34 ± 0.27
Nonanal	4.3 ± 0.69	4.54 ± 1.02	3.39 ± 0.95	4.58 ± 1.13	8.9 ± 1.23	4.74 ± 0.29	2.33 ± 0.45
(*E*,*E*)-2,6-Nonadienal	0.85 ± 0.23	0.79 ± 0.3	0.66 ± 0.14	1.23 ± 0.41	1.78 ± 0.33	1.22 ± 0.31	0.38 ± 0.04
(*E*,*Z*)-2,6-Nonadienal	51.85 ± 14.43	35.06 ± 1.89	41.18 ± 15.3	157.26 ± 46.16	146.21 ± 42.45	116.21 ± 48.17	14.22 ± 6.86
(*E*)-2-Nonenal	3.09 ± 0.58	2.58 ± 0.89	1.61 ± 0.51	6.52 ± 3.16	5.89 ± 1.4	4.49 ± 2.62	0.77 ± 0.11
Sub total C9	64.8 ± 17.5	48.48 ± 5.23	50.4 ± 17.74	175.68 ± 49.46	171.91 ± 48	130.96 ± 51.46	19.55 ± 7.68
Benzaldehyde	11.68 ± 0.51	11.04 ± 1.11	10.23 ± 1.08	10.25 ± 0.23	13.76 ± 0.07	10.91 ± 0.75	8.12 ± 0.07
(*E*,*E*)-2,4-Heptadienal	29.03 ± 7.39	33.18 ± 5.96	28.64 ± 1.13	31.49 ± 2.31	66.66 ± 6.5	26.68 ± 9.84	16.21 ± 2.86
Acetophenone	2.45 ± 0.16	2.4 ± 0.34	2.48 ± 0.33	2.26 ± 0.13	3 ± 0.09	2.1 ± 0.09	1.55 ± 0.03
(*E*,*E*)-3,5-Octadien-2-one	1.67 ± 0.35	1.8 ± 0.23	2.09 ± 0.59	1.84 ± 0.17	4.35 ± 0.71	2.33 ± 0.39	1.52 ± 0.49
4-Heptenal	4.68 ± 1.55	4.65 ± 1.41	8.02 ± 2.18	7.18 ± 2.28	10.56 ± 2.59	4.73 ± 0.78	3.16 ± 1.43
Linalool	2.22 ± 0.88	1.9 ± 0.34	2.36 ± 0.7	2.12 ± 0.53	1.97 ± 0.08	1.23 ± 0.14	1.51 ± 0.35
Methyl salicylate	0.35 ± 0.09	0.31 ± 0.04	0.4 ± 0.08	0.77 ± 0.21	0.52 ± 0.04	0.34 ± 0.08	0.12 ± 0.01
β-Cyclocitral	0.22 ± 0.05	0.19 ± 0.0SS7	0.26 ± 0.04	0.22 ± 0.06	0.78 ± 0.14	0.19 ± 0.04	0.08 ± 0.01
Undecanal	0.26 ± 0.1	0.31 ± 0.07	0.28 ± 0.11	0.23 ± 0.01	0.28 ± 0.01	0.24 ± 0.04	0.2 ± 0.03
β-Caryophyllene	ND	ND	ND	ND	ND	ND	ND
β-Ionone	0.75 ± 0.09	0.64 ± 0.18	1.1 ± 0.21	0.99 ± 0.21	4.24 ± 0.47	0.94 ± 0.08	0.42 ± 0.01
α-Curcumene	ND	ND	ND	ND	ND	ND	ND
β-Ionone epoxide	0.14 ± 0.04	0.17 ± 0.02	0.29 ± 0.1	0.19 ± 0.06	0.79 ± 0.12	0.18 ± 0.02	0.09 ± 0.04
Tetradecanal	0.26 ± 0.04	0.19 ± 0.03	0.17 ± 0.05	0.32 ± 0.12	0.39 ± 0.07	0.25 ± 0.03	0.23 ± 0.11
(9*E*,12*E*,15*E*)-9,12,15-Octadecatrienal	0.1 ± 0.04	0.17 ± 0	0.13 ± 0.05	0.19 ± 0.06	0.38 ± 0.05	0.14 ± 0.01	0.11 ± 0.03
Other Total	53.8 ± 8.51	56.94 ± 7.1	56.44 ± 3.5	58.04 ± 3.71	107.68 ± 9.9	50.26 ± 9.79	33.31 ± 3.55
Total	1181.16 ± 182.58	1709.79 ± 180.39	762.78 ± 40.35	2109 ± 692.11	5535.46 ± 71.23	2534.14 ± 233.14	411.73 ± 65.17

ND means not detected. Data represent the mean ± SD of three independent biological determinations.

Table S2. mRNA expression folds of CsLOXs in 9930 and other 6 lines during N-deficient treatments.

Sample & Treatment	*CsLOX1*	*CsLOX2*	*CsLOX4*	*CsLOX8*	*CsLOX9*	*CsLOX10*	*CsLOX16*	*CsLOX17*	*CsLOX19*	*CsLOX20*	*CsLOX22*	*CsLOX23*
9930 N-1	0.44	5.71	0.21	1.79	1.71	0.79	1.47	2.08	1.93	1.84	1.18	1.46
9930 N-2	0.24	2.79	0.05	1.29	1.97	0.87	0.64	2.94	1.82	1.98	1.97	0.81
9930 N-3	0.52	4.54	1.14	1.49	1.71	0.65	0.91	2.95	1.32	1.37	1.77	0.78
9930 N-4	0.20	2.94	1.35	0.79	1.79	0.86	0.85	2.16	1.79	1.50	1.79	0.93
9930 N-5	0.23	2.11	0.34	1.55	3.19	1.02	1.26	2.30	1.80	1.21	5.01	0.83
9930 N-6	2.07	4.03	2.04	1.53	2.44	1.05	1.64	5.19	1.49	1.92	3.74	1.21
9930 N-7	1.46	0.66	1.26	1.01	1.52	0.98	2.79	0.83	1.72	1.61	2.12	1.07
A23 N-1	1.64	1.49	0.54	1.08	5.52	0.80	0.83	0.56	0.96	0.85	0.91	1.14
A23 N-2	2.08	1.11	0.73	1.20	2.53	0.77	0.82	0.57	0.81	0.70	0.99	1.01
A23 N-3	0.86	0.79	0.04	0.95	1.41	0.92	0.16	0.79	0.43	0.39	0.71	0.70
A23 N-4	1.52	1.02	0.45	2.34	3.11	1.90	0.35	1.11	0.68	1.09	1.00	1.07
A23 N-5	1.96	1.43	1.39	1.20	8.58	0.76	0.66	0.60	0.91	2.86	1.14	0.96
A91 N-1	0.87	0.94	0.53	0.70	1.53	0.73	1.38	0.79	1.02	1.08	1.46	1.24
A91 N-2	1.49	0.95	2.57	1.34	0.72	1.39	1.74	0.64	2.83	2.59	1.66	0.94
A91 N-3	0.97	1.12	0.59	0.83	1.08	1.97	1.40	0.74	2.42	2.52	2.00	1.26
A91 N-4	1.01	0.74	1.88	2.57	0.95	1.97	1.91	0.60	2.67	3.66	2.59	1.05
A91 N-5	0.67	0.76	0.93	0.59	1.03	1.12	0.28	0.76	1.32	1.24	1.43	1.18
A38 N-2	0.85	0.44	0.48	1.48	1.37	1.69	1.10	1.05	1.30	1.04	1.38	1.30
A38 N-3	1.57	0.89	1.61	2.26	0.94	2.74	0.80	1.11	1.61	1.63	1.68	1.16
A38 N-4	1.29	1.24	0.68	1.26	0.74	2.42	0.08	2.00	1.36	1.18	1.66	1.26
A38 N-5	0.93	0.31	1.71	1.82	0.66	2.49	1.04	1.36	1.17	1.45	2.29	1.17
A37 N-3	0.59	0.83	1.01	2.63	1.05	1.36	0.94	1.75	1.03	1.23	1.32	1.20
A37 N-4	0.54	0.57	1.14	0.93	0.85	0.65	1.94	0.95	1.90	2.08	1.17	1.49
A37 N-5	0.68	0.54	0.55	4.21	0.88	1.89	3.71	1.21	2.31	3.15	2.05	1.18
A62 N-3	1.03	0.83	0.89	1.22	1.43	0.83	1.10	0.59	1.02	1.05	1.24	1.76
A62 N-4	1.29	0.95	0.66	1.38	1.35	1.00	0.35	0.97	1.06	0.99	1.25	1.26
A62 N-5	0.63	0.70	0.60	0.89	1.20	1.65	0.23	0.81	1.21	1.36	1.67	1.09
A74 N-3	0.72	0.49	1.24	1.33	0.65	0.47	0.54	0.53	1.10	1.10	1.20	0.98
A74 N-4	1.18	0.63	1.18	1.16	0.65	0.97	1.21	0.62	1.10	1.12	1.00	1.05
A74 N-5	0.99	0.40	1.52	0.88	0.70	0.68	0.47	0.89	0.93	0.99	1.04	0.83

Values are means of at least three replicates.

Table S3. Identification and quantification of volatiles during N-sufficient/deficient treatment in six inbred cucumber lines.

Compounds Name	A23	A37	A38	A62	A74	A91
	**Nitrogen Sufficient Treatment**					
Nonanal	149.36 ± 13.89	160.31 ± 25.67	174.61 ± 7.1	196.89 ± 6.12	174.1 ± 23.71	166.29 ± 13.3
(*E*,*E*)-2,4-Hexadienal	139.21 ± 27.83	65.04 ± 7.38	155.86 ± 19.87	94.83 ± 30.84	121.91 ± 37.54	114.78 ± 11.65
(*E*)-2-Hexenal	3805.94 ± 1711.28	12,679.04 ± 1887.02	10,237.23 ± 3486.51	9282.07 ± 6634.16	7697.82 ± 3087.99	4177.77 ± 1517.99
Hexanal	710.44 ± 102.77	471.08 ± 52.01	1188.07 ± 312.38	1249.41 ± 916.16	615.5 ± 188.79	523.46 ± 86.52
(*Z*)-6-Nonenal	64.78 ± 1.28	43.51 ± 16.12	60.58 ± 27.19	237.71 ± 32.07	92.09 ± 40.25	37.45 ± 4.71
(*E*,*Z*)-2,6-Nonadienol	4.26 ± 0.59	3.8 ± 0.78	4.52 ± 1.5	5.29 ± 0.8	4.55 ± 0.53	2.77 ± 0.2
(*E*,*Z*)-2,6-Nonadienal	174.9 ± 9.85	53.42 ± 8	74.94 ± 24.99	243.23 ± 80.52	66.25 ± 7.68	54.32 ± 6.76
Total C6 aldehydes	4655.59 ± 1640.14	13,215.16 ± 1835.89	11,581.16 ± 3817.51	10,626.31 ± 7523.49	8435.23 ± 3313.97	4816.01 ± 1604.75
Total C9 aldehydes	389.03 ± 11.54	257.24 ± 49.72	310.13 ± 41.74	743.73 ± 168.68	332.45 ± 65.58	258.06 ± 24.72
Total volatiles	5048.88 ± 1652.17	13,476.2 ± 1785.43	11,895.81 ± 3780.68	11,375.32 ± 7354.69	8772.23 ± 3377.37	5076.84 ± 1629.58
	**Nitrogen Deficient Treatment**					
Nonanal	137.39 ± 4.68	223.01 ± 22.77	198.42 ± 22.4	240.98 ± 12.73	268.52 ± 128.28	155.89 ± 6.7
(*E*,*E*)-2,4-Hexadienal	136.69 ± 17.71	308.82 ± 37.77	140.01 ± 24.28	228.76 ± 50.1	353.96 ± 252.18	169.29 ± 2.54
(*E*)-2-Hexenal	9240.65 ± 776.13	26,580.88 ± 2526.53	20,926.22 ± 1273.01	18,018.32 ± 325.85	15,641.82 ± 5257.74	13,699.03 ± 2484.23
Hexanal	341.71 ± 85.81	1321.61 ± 89.6	1019.84 ± 52.26	2342.39 ± 170.7	1717.24 ± 677.43	708.2 ± 55.2
(*Z*)-6-Nonenal	55.44 ± 11.26	179.15 ± 86.09	158.56 ± 42.66	184.74 ± 13.36	86.62 ± 55.72	85.18 ± 17.35
(*E*,*Z*)-2,6-Nonadienol	76.25 ± 6.79	6.19 ± 0.09	4.2 ± 0.73	6.88 ± 0.78	5 ± 2.01	3.94 ± 0.7
(*E*,*Z*)-2,6-Nonadienal	373.38 ± 33.42	137.81 ± 12.48	216.53 ± 84.51	251.97 ± 28.56	211.44 ± 260.05	180.87 ± 72.49
Total C6 aldehydes	9719.05 ± 731.29	28,211.31 ± 2538.24	22,086.07 ± 1238.64	20,589.47 ± 508.14	17,713.02 ± 6145.41	14,576.52 ± 2540.21
Total C9 aldehydes	566.21 ± 26.39	539.96 ± 105.59	573.52 ± 106.01	665.7 ± 36.69	566.58 ± 430.29	421.94 ± 60.78
Total volatiles	10,361.51 ± 741.93	28,757.46 ± 2434.61	22,663.79 ± 1343.12	21,262.05 ± 524.83	18,284.59 ± 6006.84	15,002.4 ± 2548.41

Values are means of at least three replicates ± SD.

Table S4. Content in macroelements of the two different nutrient solutions.

Ions Name	Nitrogen Treatments	
	**Nitrogen Sufficiency (12 mM)**	**Nitrogen Deficiency (0 mM)**
pH	5.80~6.00	
CE (mS·cm^{-1})	<3.0	
Ca^{2+} (mmol·L^{-1})	4	
NH_4^+ (mmol·L^{-1})	1	0
K^+ (mmol·L^{-1})	6	7
NO_3^- (mmol·L^{-1})	11	0
PO_4^{3-} (μmol·L^{-1})	1	
SO_4^{2-} (μmol·L^{-1})	3.5	5503.5
Mg^{2+} (mmol·L^{-1})	2	
Fe^{3+} (μmol·L^{-1})	79.01	
Mo^{6+} (μmol·L^{-1})	0.11	
Cu^{2+} (μmol·L^{-1})	0.32	
Zn^{2+} (μmol·L^{-1})	0.77	
Mn^{2+} (μmol·L^{-1})	9.59	
B^{3+} (μmol·L^{-1})	46.26	
